# Production of Thermostable Organic Solvent Tolerant Keratinolytic Protease from *Thermoactinomyces* sp. RM4: IAA Production and Plant Growth Promotion

**DOI:** 10.3389/fmicb.2016.01189

**Published:** 2016-08-05

**Authors:** Amit Verma, Hukum Singh, Mohammad S. Anwar, Shailendra Kumar, Mohammad W. Ansari, Sanjeev Agrawal

**Affiliations:** ^1^Department of Biochemistry, G. B. Pant University of Agriculture and TechnologyPantnagar, India; ^2^College of Basic Science & Humanities, Sardarkrushinagar Dantiwada Agricultural UniversityPalanpur, India; ^3^Climate Change and Forest Influence Division, Forest Research InstituteDehradun, India; ^4^Department of Biotechnology, Bheemtal Campus, Kumaun UniversityNainital, India; ^5^Forest Pathology Division, Forest Research InstituteDehradun, India; ^6^Department of Botany, Zakir Husain Delhi College, University of DelhiNew Delhi, India

**Keywords:** keratinase, *Thermoactinomyces*, ecosystem health, feather degradation, organic solvent stability, indole-3-acetic acid production

## Abstract

There are several reports about the optimization of protease production, but only few have optimized the production of organic solvent tolerant keratinolytic proteases that show remarkable exploitation in the development of the non-polluting processes in biotechnological industries. The present study was carried with aim to optimize the production of a thermostable organic solvent tolerant keratinolytic protease *Thermoactinomyces* sp. RM4 utilizing chicken feathers. *Thermoactinomyces* sp. RM4 isolated from the soil sample collected from a rice mill wasteyard site near Kashipur, Uttrakhand was identified on the basis of 16S rDNA analysis. The production of organic solvent tolerant keratinolytic protease enzyme by *Thermoactinomyces* sp. RM4 was optimized by varying physical culture conditions such as pH (10.0), temperature (60°C), inoculum percentage (2%), feather concentration (2%) and agitation rate (2 g) for feather degradation. The result showed that *Thermoactinomyces* sp. RM4 potentially produces extra-cellular thermostable organic solvent tolerant keratinolytic protease in the culture medium. Further, the feather hydrolysate from keratinase production media showed plant growth promoting activity by producing indole-3-acetic acid itself. The present findings suggest that keratinolytic protease from *Thermoactinomyces* sp. RM4 offers enormous industrial applications due to its organic solvent tolerant property in peptide synthesis, practical role in feather degradation and potential function in plant growth promoting activity, which might be a superior candidate to keep ecosystem healthy and functional.

## Introduction

Enzyme catalyzed processes are attractive over chemical processes involving harsh chemicals due to their wide range of industrial applications and pollution free process. They are better options in organic synthesis, pharmaceuticals, leather processing, food production, and detergent industry (Adrio and Demain, 2014). Enzymes, especially proteases constitute an important class of industrial enzymes utilized in various commercial purposes (Verma et al., 2014). Fibrous proteins such as horn, feather, nails and hair are abundantly available in nature as wastes that can be converted to useful biomass, protein concentrate or amino acids using proteases from certain microorganisms (Anwar and Saleemuddin, 1998; Villa et al., 2013). Keratinases are proteases which are capable of degrading feather (Gupta and Singh, 2014). Various bacteria, actinomycetes, and fungi are known to produce keratinases that degrade keratinous waste found in nature (Brandelli et al., 2010). Keratinolytic microbes have become paramount important among the scientific interest owing to their capacity to produce specific keratinases and their subsequent significance in keratinic waste management (Laba et al., 2015; Wang et al., 2015). In the present decade, keratinase research has gained momentum because of its additional industrial and biotechnological applications other than those in the conventional sectors of proteases (Gupta and Ramnani, 2006; Tork et al., 2016). Poultry waste which constitutes feathers, nails, etc. are rich in keratin, can be used in feed and fertilizer industry (Brandelli, 2008; Syed et al., 2009). Microbial keratinase from fungi, bacteria, and actinomycetes is less expensive than conventionally synthesized keratinase (Gopinath et al., 2015). However, a recent method of feather meal production was performed by heat treatment and high pressure which results in loss of certain heat sensitive amino acids such as methionine, lysine and tryptophan. Heat treatment also adds to non-nutritive amino acids such as lysinoalanine and lanthionine (Brandelli and Riffel, 2005). Alternate methods include enzymatic and/or fermentation methods produce feather meal rich in rare amino acids such as cysteine, serine and proline, which can be applied as feed substrate. It can also be used in fertilizers, glues, and biodegradable films (Brandelli, 2008). It has been proposed that keratinase acting on β-pleated structure of feather keratin has prospective usage in dissolution of prion plaques (Gupta and Ramnani, 2006; Tiwary and Gupta, 2010). Enzymatic processes in non-aqueous media are limited by the specificity and instability of the enzymes in the presence of organic solvents, where the reaction occurs in organic media. Organic solvent stable enzymes showing increased activity and stability in non-aqueous media are in large demand for their increasing application in organic synthesis (Patil et al., 2014). Applications of these enzymes vary widely from food additives, flavors and fragrances to pharmaceuticals, pesticides and specialty polymers (Sheldon, 1996; Gopinath et al., 2015). There are many reports regarding the production of proteases, however, the literature revealed a paucity of information about organic solvent tolerant keratinolytic proteases.

Here, we report the optimization of different physical factors for production of a thermostable keratinolytic protease of organic solvent stability from a strain of *Thermoactinomyces* sp. RM4 that shows >90% degradation of feather keratin within 24 h by the dissolution of the shaft. Feather degradation and keratinase production were studied simultaneously at a regular interval. The IAA production and *in vitro* assay of IAA for plant growth promotion in relation to feather hydrolysate production was performed to testify its agricultural utility.

## Materials and Methods

### Ethics Statement

We have not sacrificed any animal and live human subjects. We have not taken any live material which not raises any ethical issue. The chicken feathers we have collected from poultry dump yard side, hairs and nails we have collected from the barber shop. Therefore, the study does not have any ethical issue and an ethics review process is not needed for our study.

### Bacterial Strain

*Thermoactinomyces* sp. RM4 producing keratinolytic protease was isolated from soil sample from a rice mill wasteyard site, near Kashipur, Uttrakhand, India through enrichment method (Verma et al., 2011). *Thermoactinomyces* sp. RM4 was maintained on a nutrient agar medium of pH 9.0, stored at 4°C in the form of glycerol stock and subcultured monthly (Verma et al., 2011). Phylogenetic analysis based on 16S rDNA sequence analysis showed a high level of homology with *Thermoactinomyces* and the sequence was submitted to GenBank under the accession number HQ 705762.

### Culture Media and Protein Assay

The culture medium contained (g/L) NaCl 0.05, KH_2_PO_4_ 0.40, K_2_HPO_4_ 0.40, MgSO_4_ 0.04, FeCl_3_ 0.01 in basal salt media along with native chicken feathers 10.0 were used to determine keratinase production at pH 9.0. The protein content in culture filtrate and crude enzyme (supernatant) was determined via adopting the method as described by Lowry et al. (1951).

### Keratinase Assay

Keratinase activity was determined by modified method of Letourneau et al. (1998), using keratin as substrate. The keratin was suspended in Tris HCl buffer (0.05 M, pH 10.0) at concentration of 4 mg/mL. The reaction mixture contained 1 mL of enzyme and 1 mL of substrate solution. The sample was incubated at 60°C for 1 h with regular shaking. After incubation, the reaction was terminated with 2 mL of 10% TCA followed by centrifugation at 5000 × *g* for 15 min to remove unutilized substrate. The supernatant was measured for release of azo dyes at 595 nm against a control having enzyme and buffer only. One unit of keratinase (1 KU) was defined as the amount of enzyme which increases absorbance by 0.01 between sample and control at 595 nm in an hour under the specified conditions.

### Protease Assay

Proteolytic activity was assayed by modified method of Kunitz (1947) using 0.5% casein as substrate dissolved in 50 mM Glycine NaOH buffer pH 10.0. The reaction mixture was incubated at 80°C for 30 min, and reaction was stopped using 10% TCA. Tyrosine released was estimated using Folin Ciocalteau’s reagent and absorbance taken at 670 nm. One unit of protease (1 PU) was defined as amount of enzyme required to release 1 μg of tyrosine under the assay condition when reaction was incubated for 1 min.

### Effect of Various Environmental Conditions on Feather Degradation/Keratinase Production

Production of Keratinase and feather degradation by RM4 was studied using keratinase production media under different environmental conditions, i.e., Temperature, 45–70°C with increment of 5°C; pH 7.0–12.0 with increments of one unit, Feather Concentration, 1–5% with increment of 1%; Inoculum density, 2–10% (v/v) of cell density with increment of 2% and agitation rate, 1.75–4.48 g with increment of approximately 1.00 g along with static.

### Scanning Electron Microscopy

To characterize the degradation of feather, digested and undigested feather samples were dried and coated with gold palladium using Twin Coater JEC-550 (JEOL TECHNIC LTD., Tokyo, Japan). SEM was accomplished using a JEOL JSM-6390 microscope (Jeol Technic Ltd, Tokyo, Japan) at an accelerating voltage of 8–20 kV.

### Enzyme Purification

The crude enzyme was concentrated by ammonium sulfate precipitation method (80% saturation, w/v). The precipitates obtained were suspended in a minimum volume of 0.1 M Glycine NaOH buffer (pH 10.0). Further purification was done by a step of PEG concentration which was further applied to a Sephadex G-75 column (1 cm × 30 cm) equilibrated with 0.1 M Glycine-NaOH buffer (pH 10.0) and was eluted at a flow rate of 60 mL/h. Fractions of 1.5 mL were collected and were analyzed for protein contents (Abs 280 nm) as well as protease activity. The active fractions were pooled and used for further characterization.

### Enzyme Characterization

Sodium dodecyl sulfate polyacrylamide gel electrophoresis was performed to estimate the purity and molecular weight of the partially purified protein using 5% stacking gel and 12% resolving gel according to the method of Laemmli (1970) and Friedrich and Antranikian (1996). Electrophoresis was performed with 15 mA fixed current. Molecular weight was estimated by comparing the relative mobility of proteins of different molecular size using a standard molecular weight marker that purchased from Genei, Bangalore, India.

Sodium dodecyl sulfate polyacrylamide gel electrophoresis was performed by the method as described by Nam et al. (2002) and Kazan et al. (2005) using a 5% (w/v) stacking gel and a 12% (w/v) resolving gel. Electrophoresis was achieved at a steady 100 V for 90 min in Tris-glycine buffer, pH 8.3 (25 mM Tris-HCl, 192 mM glycine, 1 g L^-1^ SDS). The samples of enzyme after denaturing via boiling for 3 min in the presence or absence of 5 mM PMSF were loaded onto the stacking gel and were imagined by dye staining. To prepare a zymogram, SDS PAGE was carried out by adopting as described by Kazan et al. (2005). A 12% (w/v) resolving gel containing 1% casein was used in electrophoresis under non-denaturing conditions. The gel was then rinsed in 0.25% Triton X 100 for 1 h to remove SDS and further incubated for 1 h at 60°C in 0.05 M Glycine NaOH buffer, pH 9.0 for proteolysis of casein. Afterward, the gel was stained with 0.2% Commassie Brilliant Blue G250 solution. A clear zone/hydrolyzing area on the destained gel indicate the presence of alkaline protease activity.

To study the optimum pH of enzyme activity the buffer of different pH values from 4.0 to 12.0 were used. Buffers used were 0.1 M Citrate-phosphate buffer (pH 5.0–6.0), 0.1 M Tris-HCl buffers (pH 7.0–8.0) and 0.1 M Glycine-NaOH buffer (pH 9.0–12.0). The reaction mixture was assayed and the enzyme activity was determined. Effect of pH on enzyme stability was carried out by incubating the enzyme solution at different pH range of 6.0–10.0 for 2 h. The reaction mixture was assayed and the residual enzyme activity was determined.

The optimum temperature of the purified enzyme was determined by incubating the reaction mixture at different temperature ranging from 60 to 100°C. The reaction mixture was assayed, and the enzyme activity was determined. Enzyme was incubated at different temperatures ranging from 70 to 90°C. After incubation for different time periods, i.e., for 1 and 2 h, the reaction mixtures were assayed and the residual enzyme activity was determined.

The effect of various metal ions (Pb^+^, Na^+^, K^+^, Zn^2+^, Mg^2+^, Co^2+^, Mn^2+^, Mg^2+^, Ca^2+^, Ba^2+^, Cu^2+^ and Hg^2+^) on protease activity was examined for further characterization of this enzyme. The reaction was carried out with purified enzyme in the presence of different concentrations of metal ions (0.1, 1.0, and 5 mM). The enzymatic activity without metal ions served as control and was considered as 100% activity.

To investigate the effect of various chemicals such as metal chelators, surfactants, and inhibitors on protease activity, the enzyme assay was performed with purified enzyme in the presence of different concentrations (0.1, 1.0, and 5 mM) of chemicals *viz.*, PMSF, DTT, Urea, EDTA, βME, Triton X 100, Tween 20, IAA, pCMB, 1–10 Phenanthroline and SDS. The enzyme activity without chemicals served as control and was considered as 100% activity.

Substrate specificity of the enzyme was tested for a broad range of substrates (0.4% w/v) such as keratin, casein, BSA, gelatin as soluble substrate and human nails, chicken feather and hairs as insoluble substrate. The insoluble substrate was crushed to make powder to facilitate reaction. Reaction was carried with normal enzyme assay protocol with keratin replaced by other substrate.

The K_m_ and V_max_ values of the protease were calculated by using a Line weaver- Burk plot using different concentrations of keratin (0.2–2%, w/v) as substrate in enzyme assay and by plotting the values of 1/V as a function of 1/[S].

To check the stability of enzyme in organic solvents, three milliliters of crude protease was incubated with 1.0 mL of organic solvent at 60°C with a constant shaking at 3 g for 30 min. The protease activity was measured after 30 min using casein as substrate. For control, the solvent was replaced by distilled water. The organic solvents chosen in this study were butanol, hexane, DMSO, 2-propanol, acetone, toluene, ethanol, xylene, pyridine, and benzene.

### *In vitro* Screening of Plant Growth Promoting Activities

Production of IAA was investigated in optimal Keratinase production medium with different concentrations of L-tryptophan (0, 0.02, 0.04, 0.06, 0.08, and 0.1%). Cells were collected by centrifugation at 11963 *g* for 25 min at 4°C. IAA in the supernatants was estimated by mixing 4 ml Salkowski reagent (1 ml of 0.5 M FeCl_3_ dissolved in 50 ml of 35% HClO_4_) with 1 ml culture supernatant followed by measuring absorbance at 530 nm after 30 min.

Further, the feather hydrolysate was testified for its effect on seed germination and plant growth. The experiment was carried out in tea cups with autoclaved dried mud and Gram (*Cicer arietinum*) seeds as material. The feather hydrolysate was added 1 ml/g of soil for seed germination and further in 1:4 along with water necessary for plant growth in test and only water in control. The experiment was carried out for 2 weeks.

### Statistical Analysis

For statistical analysis, a standard deviation for each experimental result was calculated using the Excel Spreadsheets available in the Microsoft Excel and graphs were prepared using ORIGIN software Version 6.

## Results

### *Thermoactinomyces* sp. RM4: Culture Conditions for Keratinase Production and Feather Degradation with Respect to pH, Temperature, Inoculum Percentage, and Feather Concentration

**FIGURE 1:**
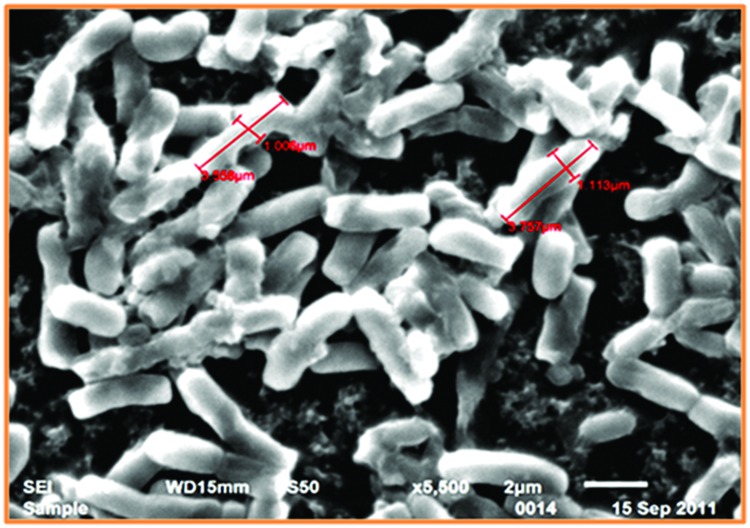
**Scanning electron microscopy of *Thermoactinomyces* sp. RM4**.

**FIGURE 2:**
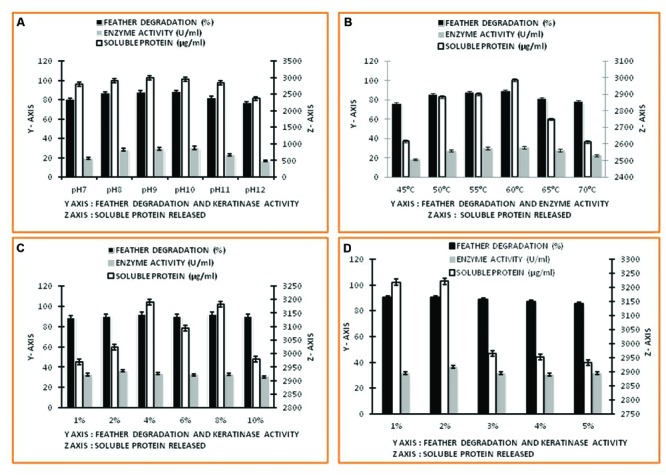
**Factors affecting keratinase enzyme production and feather degradation in keratinase production media. (A)** Effect of pH; **(B)** Effect of temperature; **(C)** Effect of inoculum percentage; **(D)** Effect of feather concentration.

The SEM study of *Thermoactinomyces* sp. RM4 showed that its size ranged from 1.006 to 3.757 μM (**Figure 1**). The pH ranged between 7.0 and 10.0 of the medium was observed to greatly affect feather degradation, soluble protein, and keratinase production (**Figure 2A**). Feather degradation, soluble protein, and keratinase production was found to be effected by initial media pH. The optimum pH for feather degradation and keratinase production was 10.0 and well within the range of pH 8.0–11.0 (**Figure 2A**). Owing to the above observations, enzyme under study can be classified as an alkaline protease and was most active under neutral or basic conditions (**Figure 2A**). In addition, the feather degradation, soluble protein, and keratinase production of *Thermoactinomyces* sp. RM4 were optimal at an incubation temperature of 60°C (**Figure 2B**). At higher and lower temperature a decrease in all the three factors, i.e., feather degradation, soluble protein, and keratinase production were observed. All the three factors were found to be well within the temperature range of 50–60°C (**Figure 2B**). Besides, inoculum size slightly affects feather degradation, soluble protein, and keratinase production (**Figure 2C**). Further, the increasing inoculum size (from 1 to 4%) showed improved feather degradation and soluble protein content except keratinase production doesn’t varied and maximum keratinase production achieved at 2% inoculum alongwith comparable feather degradation (**Figure 2C**). Optimum feather concentration for feather degradation, soluble protein and keratinase production was found to be at 2% in submerged condition (**Figure 2D**).

### Effect of Agitation Rate on Feather degradation and Keratinase Production and Scanning Electron Microscopy to Evaluate Breakdown of the Regular Structure

**FIGURE 3:**
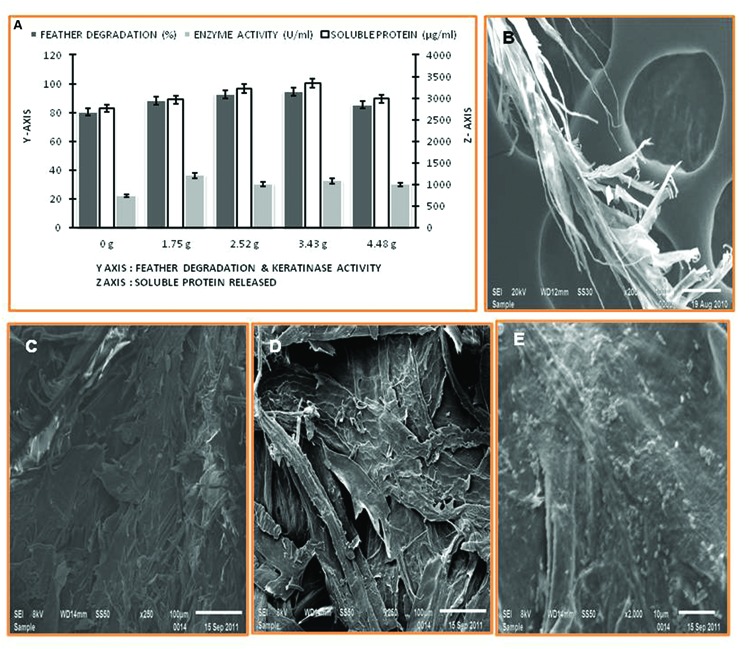
**Feather degradation, keratinase activity, and soluble protein released under different rates of agitation, and SEM of degraded feather. (A)** Effect of agitation rate; **(B–E)** SEM of feather degradation in keratinase production media.

The percentage of feather degradation and keratinase production show inversely proportionality to the feather concentration in cultivation media. The rotation speed of 1.75 g yielded maximum keratinase production, however, feather degradation was comparably higher at 3.43 g (**Figure 3A**). Keratinolytic enzyme production by the *Thermoactinomyces* strain RM4 led to breakdown of the regular structure of feather keratin fibers, which was visualized using SEM (**Figures 3B–E**).

### Keratinase Enzyme Purification

**FIGURE 4:**
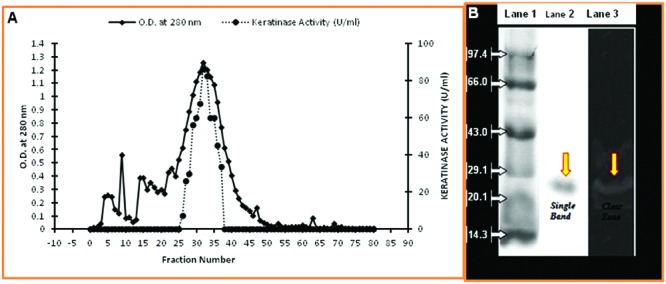
**Purification of keratinase from *Thermoactinomyces* sp. RM4. (A)** Elution profile of keratinase of *Thermoactinomyces* sp. strain RM4 in Sephadex G-75 column; **(B)** SDS–PAGE assay and the activity of purified Keratinase from *Thermoactinomyces* sp. RM4. Lane1: standard protein markers; Lane 2: purified proteases; Lane 3: zymography of purified enzyme.

In the present study, the keratinolytic protease was purified using a step of ammonium sulfate concentration method followed by PEG concentration. The concentrated enzyme was further purified by Sephadex G-75 column, and peak was obtained at fraction number 32–35 (**Figure 4A**). Enzyme purification was up to 2.51 fold (specific activity of 224.13 U/mg), and approximately 64.6% yield was achieved (**Table 1**). The purified enzyme showed a single protein band on SDS-PAGE which confirms the homogeneity of purified keratinase obtained in the above purification steps. The molecular mass of the protease estimated from the relative mobility of the standard protein on SDS-PAGE was between 20 and 29 KD. Zymogram analysis also showed a prominent activity band between ranges of 20 and 29 KD and confirms the molecular weight of enzyme approximately to 25 KD (**Figure 4B**).

**Table 1:** Purification of the keratinolytic protease from *Thermoactinomyces* sp. strain RM4.

Purification step	Total protein (mg)	Total activity (U)	Specific activity (U/mg)	Recovery (%)	Fold purification
Crude	125	8500	68	100	1.0
Ammonium sulfate concentration	90.3	8040	89.0	94.5	1.31
Sephadex G 75 column	23.2	5200	224.13	64.6	2.51

### Enzyme Characterization: Keratinase Activity and Stability under Varied pH and Temperature

**FIGURE 5:**
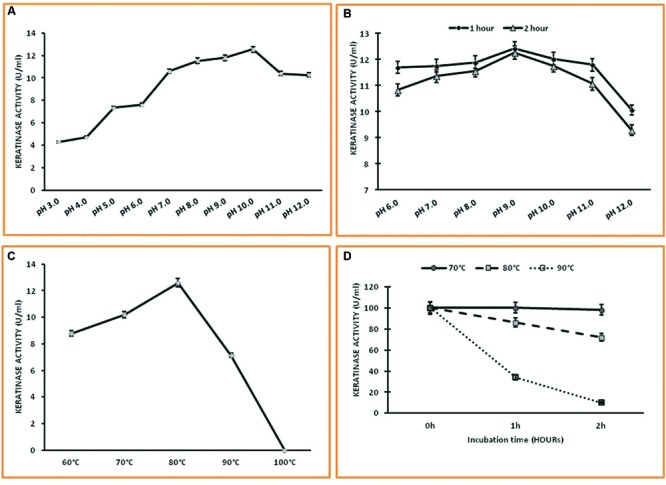
**Characterization of keratinase from *Thermoactinomyces* sp. RM4. (A,B)** Effect of pH on the activity and the stability of Keratinase enzyme; **(C,D)** Effect of temperature on keratinase activity and stability.

The enzyme was active and stable over a wide range of pH 6.0–12.0 with optimum activity at pH 10.0 which is different from earlier reports (**Figures 5A,B**). It was completely stable in the pH range of 6.0–11.0 for 1 h and shows remarkable pH stability for 2 h in the pH range of 6.0–10.0 with notable loss of enzyme activity at higher pH, i.e., 12.0. The temperature profile of Keratinase activity from *Thermoactinomyces* sp. RM4 was presented in **Figures 5C** The maximum enzyme activity was obtained at 80°C and works well within the range of 60–80°C. The keratinase of *Thermoactinomyces* sp. RM4 showed 90% stability at 80°C for 1 h. The enzyme was found to be having 35% stability even at 90°C after 1 h incubation. The keratinase from this isolate showed 100% stability at 70°C even after 2 h of incubation (**Figure 5D**).

### Effect of Metal Ions, Inhibitors, Substrates, and Organic Solvent on Keratinase Activity

**FIGURE 6:**
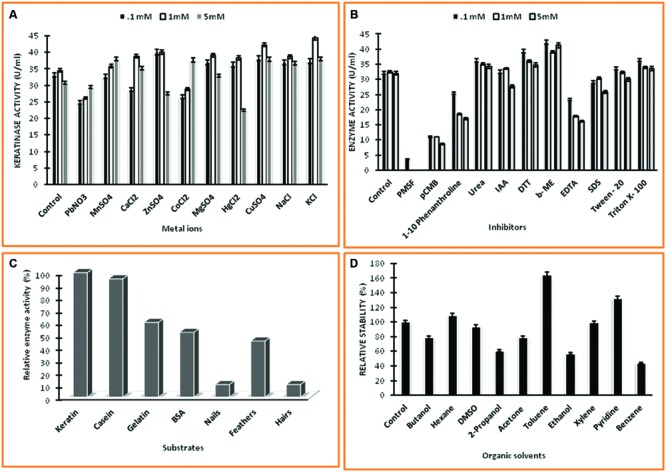
**Characterization of keratinase from *Thermoactinomyces* sp. RM4. (A)** Effect of different metal ions on keratinase enzyme activity at different concentrations of 0.1, 1.0, and 5.0 mM; **(B)** Effect of different chemicals on enzyme activity at different concentrations of 0.1, 1.0, and 5.0 mM; **(C)** Substrate specificity of keratinase; **(D)** Effect of organic solvents on enzyme activity.

Several metal ions at three concentrations *viz.*, 0.1, 1.0, 5.0 mM were assayed for their effects on keratinase of *Thermoactinomyces* sp. RM4 (**Figure 6A**). The activity of the enzyme was essentially affected by monovalent cations (Na^+^ and K^+^), and these metal ions showed an increment in enzyme activity at all of their concentrations testified. Keratinase activity enhanced with the addition of Cu^2+^, Mg^2+^, and Mn^2+^ at defined concentrations as compared to the control. This result indicates that the enzyme required Cu^2+^, Mg^2+^, and Mn^2+^ for optimal activity. Furthermore, the enzyme was slightly activated by some metal ions at their lower concentrations viz. Zn^2+^, Hg^2+^ and underwent inhibition at higher concentration. Some metal ions viz. Pb^2+^, Co^2+^, and Ca^2+^ moderately inhibited keratinase activity at their lower concentration but activation at their higher concentrations (**Figure 6A**). On the other hand, the effects of various chemicals such as inhibitors, detergents, surfactants, reducing agents on keratinase activity at three concentrations *viz.*, 0.1, 1.0, 5.0 mM, which are shown in **Figures 6B** The PMSF completely inhibited the enzyme activity at all of its used concentrations, thus confirming enzyme’s serine protease nature. Partial inhibition of enzyme activity was observed with pCMB and EDTA at all of their used concentration Remarkable decrease in the enzyme activity has been observed in case of EDTA with its increasing concentration. The enzyme activity increased significantly with addition of non-ionic detergents Triton-X, Tween 20 and reducing agents DTT, β-ME, however, enzyme activity decreased in case of phenanthroline, SDS and IAA. In case of phenantroline and SDS the enzyme activity decreased at all concentrations especially at higher concentrations, i.e., 1.0 and 5.0 mM. Contrastingly, IAA doesn’t reflect significant decrease in enzyme activity at 0.1 and 1.0 mM but at 5.0 mM approximately enzyme activity decremented by 20% as compared to control. The relative hydrolysis rates of various substrates were investigated to elucidate substrate specificity of keratinase from *Thermoactinomyces* sp. RM4 (**Figure 6C**). The K_m_ and V_max_ values of Keratinase alongwith other properties was s in the **Table 2** The organic solvent (at a final concentration of 25% (v/v)) stability of protease from *Thermoactinomyces* sp. RM4 was observed in the following order: toluene (*log P 2.5*) > pyridine (*log P 0.71*) > n-hexane (*log P 3.5*) > xylene (*log P 3.1*) > DMSO(*log P -1.378*) > ethanol (*log P -0.235*) > butanol (*log P 0.8*) = acetone (*log P -0.24*) > 2-propanol(*log P 0.074*) > benzene (*log P 2.0*) (**Figure 6D**).

**Table 2:** Properties of keratinase from *Thermoactinomyces* sp. strain RM4.

S. no.	Biochemical property	Value
1	pH optima	10.0
2	Temperature optima	80°C
3	Molecular weight	25 KD
4	Substrate specificity	Keratin
5	K_m_	20 μg/mL ± 0.05
6	V_max_	400 μg/min/mL ± 10
7	K_cat_	1.73913 ± 0.043
8	k_cat_/k_m_	0.08695 ± 0.0021

### *In vitro* Screening of Plant Growth Promoting Activities

**FIGURE 7:**
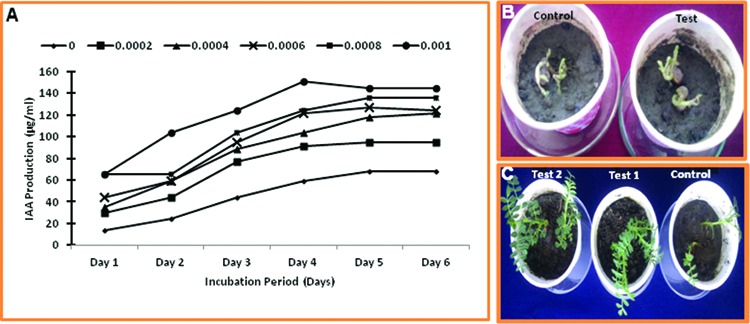
**Plant growth promoting activity of feather hydrolysate from keratinase production media. (A)** Indole-3-acetic acid (IAA) production in culture media supplemented with different concentrations of L- tryptophan during incubation of 6 days; **(B)** Effect of feather hydrolysate supplementation in soil on seed germination; **(C)** Effect of feather hydrolysate supplementation in soil on plant growth.

The production of IAA was increased with increasing concentration of L-tryptophan, and maximum production of IAA (150.70 ± 7.9 μg/ml) was observed after 4 days of cultivation in the optimal feather medium supplemented with 0.1% L-tryptophan. Even in the optimal feather medium without L-tryptophan supplementation, IAA was also produced to comparable concentration (**Figure 7A**), which provide the basis of use of feather hydrolysate from *Thermoactinomyces* sp. RM4 to be utilized in the fertilization process of soil. The application of feather hydrolysate was given in soil to evaluate seed germination of and plant growth and development of *Cicer arietinum* (**Figures 7B,C**). Seed germination was early in case of soil supplemented with feather culture lysate in comparison to control (**Figure 7B**). Plant growth was higher under test condition in comparison to control (**Figure 7C**).

## Discussion

Keratinases, the proteolytic enzymes that mainly active upon keratin substrates, break disulfide bridges present in the complex structure of keratin to give rise to simplified forms. Keratinases are vital in recent research of industrial applications including animal nutrition, protein synthesis and its supplements, leather fabricate, textile practices, detergent formulation, feather utilization, the pharmaceutical research, and waste management (Gopinath et al., 2015). Several studies showed that keratinolytic enzyme from bacterial species cultured in medium containing chicken feather show various applications in waste feathers management and in detergents formulation (Fakhfakh-Zouari et al., 2010; Fang et al., 2016). In the present study, a feather-degrading bacterial strain *Thermoactinomyces* sp. strain RM4 was used to produce keratinase under submerged fermentation (SmF) condition. Here, keratinase shows differential activity under treatment of detergents. Apart from keratinase production, total soluble protein released in addition to feather degradation was observed during SmF utilizing one variable at a time by employing strategy representing one of a common way for standardizing enzyme production (Gupta and Ramnani, 2006). Bacterial culture is capable of utilizing the chicken feathers as sole carbon and nitrogen source (Jeong et al., 2010). Feather degradation started from its tiny entity, i.e., barbules and the barbs, fibers along with the shaft region also digested by bacteria (Laba et al., 2015). Here, keratinase from *Thermoactinomyces* strain RM4 is thermostable and organic solvent tolerant, which has potential role in feather degradation and plant growth promotion.

Many of the reported proteases are have their pH optima in alkaline range which makes them beneficial for various industrial purposes (Cheng et al., 1995; Suntornsuk and Suntornsuk, 2003; Rahman et al., 2005; El-Bondkly and El-Gendy, 2010). Our result shows the optimum pH for feather degradation and keratinase production was in the range of pH 8.0–11.0. These results are in line with previous reports which indicate that keratinase produced by microorganisms could be classified as an alkaline protease and was most active under neutral or basic conditions. Alkaline pH possibly favors keratin degradation as higher pH modifies cystine residues to lathionine (Frankena et al., 1986; Rissen and Antranikian, 2001; Selvam et al., 2013) to make easy keratinase action. The resulting lanthionine residue makes keratin structure vulnerable to keratinase hydrolytic action (Friedrich and Antranikian, 1996), and thus alkaline keratinases are much industrially applicable and can be applied to various purposes. In addition, at higher and lower temperature a decrease in all the three factors, i.e., feather degradation, soluble protein, and keratinase production were observed. All the three factors were found to be well within the temperature range of 50–60°C which is the temperature range for optimum growth of various actinomycetes (Brandelli et al., 2010). Further, many workers reported the feather degradation and keratinase production in the range of 50–70°C (Williams et al., 1990; Rissen and Antranikian, 2001; Hu et al., 2013; Poopathi et al., 2014). In case of extracellular enzymes temperature was found to effect the secretion which can be due to changing of physical properties of the cell membrane (Frankena et al., 1986; Rahman et al., 2005). Apart from this a lowering in activity of enzyme occurs at high temperature due to protein denaturation (Kataoka et al., 2014). In contrast, inoculum size of culture added to media doesn’t affects feather degradation and keratinase production much in the present study. However, the repression of keratinase production at higher inoculation volume (>2% v/v) was quite comparable with some of the earlier reports, where keratinase production was higher at lower inoculation volume (Gupta and Ramnani, 2006). Tiwary and Gupta (2010) reported maximum keratinase production at 2% v/v inoculum level by one variable at a time approach. The percentage of feather degradation and keratinase production both shows inversely proportionality to the feather concentration in cultivation media. It was demonstrated that high feather concentrations may cause substrate inhibition or repression of keratinase production, resulting in a low percentage of feather degradation (Selvam et al., 2013). The high feather concentrations results to substrate inhibition which results in decrement of feather degradation (Suntornsuk and Suntornsuk, 2003). It was seen that the kinetics of keratinase production and that of keratin degradation do not overlap, and keratinolysis cannot act as indicator of keratinase production (Gupta and Ramnani, 2006; Habbeche et al., 2014).

The agitation rate is one of the important parameter to optimize in case of enzyme production both at low and pilot scale. Usually, increased rotation speed provides high oxygen transfer rate and better interaction of cell and substrate which supports better growth along with increased enzyme production. In contrast, at low rotation speed, low enzyme production and feather degradation were observed due to improper substrate accessibility. A high speed gave minimal bacterial growth, feather degradation and keratinase production because of a high shear rate generated by this speed would damage bacterial cells (Suntornsuk and Suntornsuk, 2003; Selvam et al., 2013). In SEM study, keratinolytic enzyme production by the strain RM4 causes degradation of feather structure. Bacterial cells can be seen on the feather surfaces and the structural composition of keratin fibers was highly disintegrated due to their action. The complete degradation of feathers by the strain RM4 was observed after 4 days at 60°C. These results indicate that *Thermoactinomyces* strain RM4 may be applicable in the preparation of feed additives for animal feed, production of nitrogenous fertilizers from keratinolytic waste, and it can also be relevant in the treatment of feathers as environmental pollutants creating solid waste problem (Villa et al., 2013).

Most of the reported Keratinases till date are studied from *Bacillus* species with commercially utilization reported from *Bacillus licheniformis* (Parrado et al., 2014; Gopinath et al., 2015). Scarcity of literature found in case of keratinase purification and characterization from actinomycetes especially *Thermoactinomyces.* Here, the keratinase purified from *Thermoactinomyces* sp. strain RM4 was small size of 25 KD. The molecular masses of several keratinases have been determined and were in the range from 18 KD for *Streptomyces albidoflavus* (Bressollier et al., 1999) to 240 KD in *Kocuria rosea* (Bernal et al., 2006), most keratinases have molecular weight less than 50 KD (Suntornsuk and Suntornsuk, 2003; Syed et al., 2009; Tork et al., 2016). The majority of keratinases are monomeric enzymes; however, oligomeric keratinases are also reported (Lee et al., 2012). Keratinase has been purified and characterized from different bacterium was stable in a broad range of pH (8.0–12.0) and temperature (20–50°C) in addition to optimum at around pH 10.0 and 37°C (Wang and Liao, 2014; Sanghvi et al., 2016). In the present study, keratinase was an alkaline protease showing optimal keratinolytic activity at pH 10.0. Earlier, most of the keratinases reported are active in neutral to alkaline pH ranged 7.5–10.0 (Brandelli et al., 2010; Sanghvi et al., 2016). The keratinase from this isolate was even active and stable at acidic pH. The lowest reported value of pH 4.0 is for *Streptomyces pactum* DSM40530 (Bockle et al., 1995), and the highest reported value of pH 13.0 is for *Bacillus halodurans* AH-101 (Takami et al., 1999; Prakash et al., 2010). Temperature optima reported is higher than the earlier reports. Keratinases from other bacteria showed temperature optima in the range of 40–70°C (Suh and Lee, 2001; Gessesse et al., 2003; Brandelli et al., 2010) while recombinant keratinase from *B. licheniformis* expressed in *Bacillus megaterium* had optima at 75°C (Suh and Lee, 2001). The thermostability shown by keratinase from this isolate was remarkable and much better than the previous reports from actinomycetes (Tatineni et al., 2008; Syed et al., 2009).

The effects of monovalent ions on the keratinase activity are in contrast to some previous reports but this is usually due to the effect of enzyme substrate complex stabilization and also monovalent ions helps in charge stabilization during the hydrolytic reactions (Jaouadi et al., 2013). Further report also showed that the enzymatic action of the keratinase induced by Na^+^, K^+^, Mg^2+^, Hg^2+^ (Tork et al., 2016). Enzyme activity was enhanced following the addition of Cu^2+^, Mg^2+^, and Mn^2+^ at all concentrations, as compared to the control. This result indicated that the enzyme required Cu^2+^, Mg^2+^, and Mn^2+^ for its optimal activity. The increase in protease activity with Cu^2+^, Mg^2+^, and Mn^2+^ indicates that metal ions imparts protective effect to enzyme against thermal denaturation, thereby playing a vital role in maintaining its active confirmation at higher temperature (Kumar and Takagi, 1999; Jaouadi et al., 2013). Furthermore, the enzyme was slightly activated by some metal ions at their lower concentrations *viz.*, Zn^2+^, Hg^2+^ and underwent inhibition at higher concentration. Some metal ions *viz.*, Pb^2+^, Co^2+^, and Ca^2+^ moderately inhibited keratinase activity at their lower concentration but activation at their higher concentrations. On the other hand, mostly the keratinases are classified as serine-type proteases and among actinomycetes, keratinases are reported previously to be classified as serine proteases (Bockle et al., 1995; Tork et al., 2016). In addition, the highest activity was observed with keratin followed by casein, gelatin, BSA. Hydrolytic activity against feathers, nails and hairs was also observed, which further confirms the keratinolytic nature of enzyme. Apartly, absence of collagenase activity provides further support for the utility of Keratinase from *Thermoactinomyces* sp. *RM4* in leather industry which was published in an earlier report (Verma et al., 2011). In the present study, this enzyme showed complete inhibition by PMSF that shows its serine protease nature along with pCMB inhibition. It indicates that sulfhydryl groups of cysteine residues are present at or near the active site. EDTA, a metal chelator had inhibited the enzyme activity suggesting metalloprotease nature of keratinase reported here upon. Cysteine usually not found at metalloprotease active site but if occur in vicinity results to impairment of substrate accessibility (Gupta and Ramnani, 2006). The keratinase in the present study had shown broad substrate specificity against both the soluble and complex insoluble substrates like feathers, nails and human hairs. The enzyme effectiveness for hydrolysis of insoluble substrates adds to its industrial application potential and can be utilized as safe feed additive due to its non-pathogenic bacterium source, i.e., *Thermoactinomyces* (Brandelli et al., 2010). Apartly, bacteria can be used as potent bioremediator of keratinolytic waste to put them to be utilized as fertilizer which can support the success of poultry farm as small scale industry (Anwar and Saleemuddin, 1998). Keratinase activity decreases with anionic SDS addition. Mostly the keratinases are classified as serine-type proteases. Among actinomycetes, several keratinases reported previously are classified as serine proteases (Jaouadi et al., 2013). Further, the property of solvent tolerance is strain-specific and the toxicity of a given solvent correlates with the logarithm of its partition coefficient in n-octanol and water (log *P*ow). Generally, high values of log *P* of solvent, i.e., the logarithm of the partition coefficient P of the solvent between octanol and water (Laane et al., 1987; Patil et al., 2014) results in greater stability of protease in that particular solvent (Ogino et al., 1995). Rahman et al. (2005) reported a solvent stable protease from *Bacillus pumilus* which has got stability in the presence of benzene and toluene both but contrastingly in present study, keratinase activity incremented by about 64% in case of toluene but decremented to about 44% in case of benzene. The solvent stability of enzymes varied much depending upon their bacterial source as well as their protein secondary structures at molecular level (Ogino et al., 1995; Abusham et al., 2009). However, the results in this study showed that the keratinolytic protease, from RM4 strain, was not only stable in the presence of organic solvents having Log *P* ≥ 2.0, but also in the presence of some organic solvents with the Log *P* < 2.0. These results indicated that this keratinase might be a novel solvent-stable keratinase which can be applied for non-aqueous biocatalysis. In the present study, one of the interesting results is the production of IAA in feather culture medium. Plants survive in the soil by interacting with various microbial community which helps in their growth as well as resistance to different pathogens (Anwar et al., 2014). Microbes interacts with plant roots which is popularly known as “*Rhizosphere*” region of soil and stimulates plant growth by different factors like phosphate solubilization, ammonia production, siderophore production, hydrolytic enzyme secretion, and IAA production (Jeong et al., 2010). *Thermoactinomyces* sp. strain RM4 which is producing extracellular keratinase, a hydrolytic enzyme alongwith production of IAA in the culture medium by utilizing tryptophan released by dissolution of feathers without requirement of additional tryptophan. Thus, the culture filtrate was further examined for its effect on seed germination and plant growth where it fetched positive results supporting the applicability of culture in the production of fertilizers from chicken feathers alongwith industrially relevant enzyme production. Our study shows that keratinase, pH optima of 10.0, from *Thermoactinomyces* strain RM4 provides its application in laundry detergent and in leather industries. Further, reported keratinase can also be applied for non-aqueous biocatalysis due to its organic solvent stability. Additionally, it could be a key player for sustainable agricultural production by promoting plant growth and development under varied environmental conditions.

## Conclusion

Keratinase production was optimized by using production media having chicken feathers as sole carbon and nitrogen source which removes the hurdle of huge substrate cost in industrial enzyme production. The keratinase production was accompanied with observable IAA production in the culture media even without tryptophan supplementation which paves way to utilize spent media after enzyme recovery in agriculture as slow release nitrogenous fertilizer.

Keratinase was purified and characterized biochemically. Keratinase from this RM4 strain is thermostable, organic solvent stable protease having pH optima of 10.0 and temperature optima of 80°C. Apartly, it possesses several other interesting properties which support it as a potential candidate to be utilized in laundry detergent, leather industries, non-aqueous catalysis. Agricultural applicability of spent production media after enzyme recovery as confirmed by *in vitro* experiments is an additional features which opens a new area of research for best utilization of feather hydrolysate as fertilizer.

## Author Contributions

Conceived and designed the experiments: AV and SK. Performed the experiments: AV and SA. Analyzed the data: HS and MWA. Wrote the paper: AV, HS, and MWA.

## Conflict of Interest Statement

The authors declare that the research was conducted in the absence of any commercial or financial relationships that could be construed as a potential conflict of interest.

## ABBREVIATIONS

β-ME: β-mercaptoethanol
BSA: bovine serum albumin
DMSO: dimethyl sulfoxide
DTT: dithiothreitol
EDTA: ethylene diamine tetra acetic acid
IAA: indole-3-acetic acid
pCMB: para chloro mercuric benzoate
PEG: poly ethylene glycol
PMSF: phenyl methyl sulfonyl fluoride
SEM: scanning electron microscopy
SDS-PAGE: sodium dodecyl sulfate polyacrylamide gel electrophoresis
TCA: tri-chloro acetic acid.
